# Cefiderocol Comparative Resistance and Clinical Predictors in CRE-BSI: Data from an OXA-48–Endemic Region with Rising OXA-48/NDM Coproducers

**DOI:** 10.3390/antibiotics14111057

**Published:** 2025-10-22

**Authors:** Rıdvan Dumlu, Ali Mert

**Affiliations:** 1 Department of Infectious Diseases and Clinical Microbiology, Faculty of Medicine, Istanbul Medipol University, Istanbul 34214, Turkey; 2 Department of Internal Medicine, Faculty of Medicine, Istanbul Medipol University, Istanbul 34214, Turkey; alimert@medipol.edu.tr

**Keywords:** cefiderocol, carbapenem resistance enterobacterales, drug resistance, bacteremia, carbapenemases, Turkey

## Abstract

**Background/Objectives**: Bloodstream infections (BSIs) caused by carbapenem-resistant Enterobacterales (CREs) are a growing public health threat due to limited therapeutic options and high mortality. In Turkey, oxacillinase-48 (OXA-48) producers predominate, while OXA-48/New Delhi metallo-β-lactamase (NDM) co-producers are increasingly detected. Although ceftazidime–avibactam (CAZ-AVI) is effective against OXA-48, treating NDM-positive isolates remains challenging. Cefiderocol, a novel siderophore cephalosporin active against both serine- and metallo-β-lactamases, is not yet available in Turkey. Establishing baseline susceptibility rates and identifying clinical predictors of resistance are, therefore, crucial before its introduction. **Methods**: This retrospective study included adult patients with CRE-BSIs diagnosed at a tertiary university hospital in Istanbul between January and December 2023. Demographic, clinical, and microbiological data were collected from electronic medical records. Susceptibility to cefiderocol, CAZ-AVI, and colistin was determined according to the European Committee on Antimicrobial Susceptibility Testing (EUCAST) 2023 criteria. Risk factors for cefiderocol resistance were analyzed. **Results**: Among 202 isolates, cefiderocol showed the highest susceptibility (94%, *n* = 190), followed by CAZ-AVI (82%, *n* = 166) and colistin (70%, *n* = 141), with all pairwise differences being statistically significant (*p* < 0.001). Cefiderocol resistance (6%, *n* = 12) was significantly associated with hematologic malignancy, hematopoietic stem cell transplantation, prior CAZ-AVI or polymyxin exposure, prolonged hospitalization, and repeated admissions. **Conclusions**: Cefiderocol demonstrated potent in vitro activity against CRE-BSI isolates, with resistance confined to distinct high-risk clinical settings. This pre-implementation study provides baseline microbiological and epidemiological data from an OXA-48 endemic region with rising NDM prevalence, underscoring the importance of early surveillance and stewardship strategies before cefiderocol becomes clinically available.

## 1. Introduction

Carbapenem-resistant Enterobacterales (CREs) have become a major global public health threat due to rising incidence, limited treatment options, and high mortality [1,2,3,4]. The most important resistance mechanism in CREs is carbapenemase production. The 2024 Infectious Diseases Society of America (IDSA) guidelines recommend tailoring therapy according to carbapenemase type and prioritizing newer β-lactams such as ceftazidime–avibactam (CAZ-AVI), meropenem–vaborbactam, and cefiderocol, while polymyxins are no longer considered first-line options because of their toxicity and increased mortality risks [5].

In Turkey, CRE epidemiology is largely driven by oxacillinase-48 (OXA-48) carbapenemase, although OXA-48/New Delhi metallo-β-lactamase (NDM) co-producers are increasingly reported [6]. While OXA-48 producers are generally managed with CAZ-AVI, therapeutic options for NDM co-producer isolates remain extremely limited. For these cases, the IDSA recommends CAZ-AVI and aztreonam in combination or cefiderocol [5]. However, aztreonam and cefiderocol are not currently available in Turkey, forcing clinicians to rely on polymyxin-based therapies despite their disadvantages. When resistance to polymyxins occurs, a critical therapeutic gap emerges—particularly in bloodstream infections (BSIs), where mortality is highest [7].

Cefiderocol is a novel siderophore cephalosporin that utilizes iron transport systems to enter bacterial cells and remains stable against both serine- and metallo-β-lactamases (MBL) [8,9,10]. This “Trojan horse” mechanism enables activity despite porin loss, efflux pump overexpression, or carbapenemase production, providing broad in vitro potency [11,12,13]. However, elevated minimum inhibitory concentrations (MICs) and resistance have been reported in NDM-positive isolates [14].

Cefiderocol has not yet been introduced for clinical use in Turkey, and carbapenemase typing is not routinely performed in most laboratories. Therefore, in settings where genotypic characterization is unavailable, identifying clinical predictors of resistance becomes particularly important. In this real-world pre-implementation study, we compared the in vitro activity of cefiderocol with available options (CAZ-AVI and colistin) and assessed clinical risk factors associated with cefiderocol resistance. However, as a single-center, retrospective study reporting only in vitro data without clinical outcomes, the findings should be interpreted with caution.

## 2. Results

**Figure 1:**
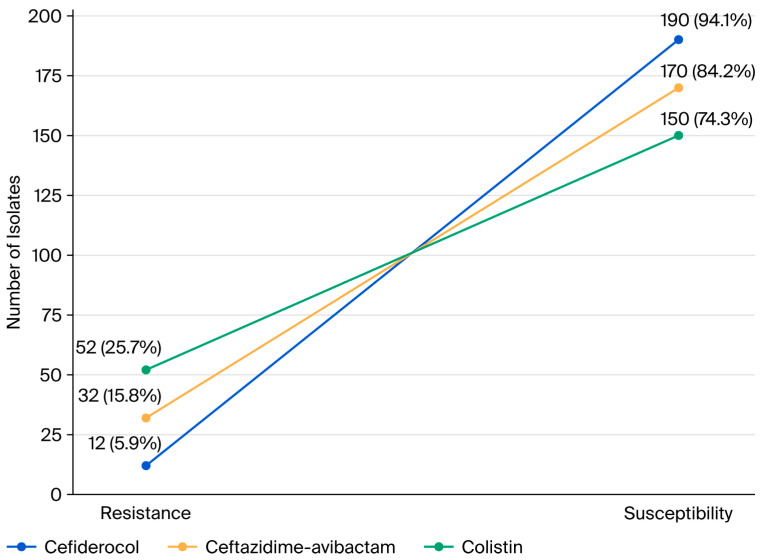
Comparative susceptibility rates of cefiderocol, ceftazidime–avibactam, and colistin among CRE-BSI isolate.

A total of 202 patients were included in the study, with a median age of 52.5 years; 52.5% (*n* = 106) of the patients were male. The susceptibility rate of cefiderocol among CRE isolates causing BSIs was 94.1% (*n* = 190). In comparison, susceptibility rates for CAZ-AVI and colistin were 84.2% (*n* = 170) and 74.3% (*n* = 150), respectively. According to McNemar’s test, cefiderocol susceptibility was significantly higher than that of both CAZ-AVI and colistin (*p* < 0.001 for both), and CAZ-AVI susceptibility was significantly higher than colistin (*p* < 0.001) (Figure 1).

Table 1 summarizes the sociodemographic and clinical characteristics of the study population. A considerable proportion of patients had high Charlson Comorbidity Index (CCI) and Pitt Bacteremia Score (PBS) values. Among chronic comorbidities, diabetes mellitus (49%), chronic kidney disease (50.5%), congestive heart failure (55%), rheumatologic disease (50.5%), neurological disease (44.6%), solid organ malignancy (22.8%), and a history of solid organ transplantation (2.5%) were documented, with no significant differences between the cefiderocol-resistant and -susceptible groups.

In contrast, hematologic malignancy (75% vs. 10.5%, *p* < 0.001) and history of hematopoietic stem cell transplantation (HSCT) (50% vs. 2.6%, *p* < 0.001) were significantly more common among resistant cases. Prior exposure to CAZ-AVI (*p* < 0.001) or polymyxins (*p* < 0.001) within 90 days, hospitalization longer than 14 days (*p* < 0.001), and hospitalization within the past year (*p* = 0.003) were also significantly more frequent in the cefiderocol-resistant group.

Table 2 presents the microbiological data. The most frequently isolated pathogen was *Klebsiella pneumoniae*, followed by *Escherichia coli*. No significant differences were observed between the cefiderocol-resistant and -susceptible groups regarding infection focus or pathogen distribution.

## 3. Discussion

In this study, cefiderocol demonstrated high in vitro activity against CRE-BSI isolates. These results align with large-scale international studies reporting susceptibility rates between 90% and 99% depending on the Clinical Laboratory Standards Institute (CLSI) and European Committee on Antimicrobial Susceptibility Testing (EUCAST) criteria [1,13,15,16]. Comparable findings have also been reported from Turkey, where cefiderocol consistently showed potent activity against CRE isolates, although higher MIC values were occasionally observed among NDM-positive strains [11,14]. Taken together, our data confirm the concordance of local epidemiology with global evidence and underscore the relevance of cefiderocol as an important option in vitro.

In our study, susceptibility rates for CAZ-AVI and colistin were assessed alongside cefiderocol. Recent reports from Turkey have documented CAZ-AVI susceptibility ranging from 77% to 93% and colistin susceptibility ranging from 48% to 75% among CRE isolates [17,18,19,20], aligning with our findings. The overall susceptibility ranking in our cohort was cefiderocol > CAZ-AVI > colistin. This order has also been reported in a multicenter study from India [12] and a large-scale investigation from France and Belgium [1], further confirming that our results are consistent with the international literature.

The superiority of cefiderocol can be attributed to its siderophore-mediated “Trojan horse” mechanism, which facilitates its active transport into bacterial cells through iron uptake systems. This strategy offers protection against common resistance mechanisms, including porin loss, efflux pump overexpression, and carbapenemase activity, making cefiderocol less susceptible to resistance compared with other β-lactams [12]. These microbiological properties provide a clear rationale for the high in vitro activity demonstrated in our study and reported across different geographic regions.

Although uncommon, the detection of cefiderocol resistance underscores the need for caution in its clinical application. In our cohort, where routine carbapenemase typing was not widely available, several clinical predictors of resistance were identified. Hematologic malignancy, prior HSCT, recent exposure to CAZ-AVI or polymyxins, prolonged hospitalization, and recurrent admissions were all significantly more frequent among cefiderocol-resistant cases.

These findings are in line with previous reports. In patients with hematologic malignancies, particularly those with acute lymphoblastic leukemia, cefiderocol-resistant *K. pneumoniae* strains have been more frequently identified, with resistance linked to combined mechanisms such as NDM production and CirA deficiency [21]. Moreover, prolonged hospitalization has been shown to increase the daily risk of acquiring infections with resistant Gram-negative bacteria by approximately 1%, especially when compounded by underlying comorbidities [22]. Data from the Centers for Disease Control (CDC) and other epidemiological studies also emphasize prolonged hospitalization, immunosuppression, and repeated antibiotic exposure as major risk factors for infections caused by resistant pathogens [23,24].

In addition, cefiderocol resistance has been associated with several mechanisms linked to prior antibiotic exposure. Reported pathways include mutations in siderophore receptors, disruption of iron uptake systems, and alterations in efflux pump regulation [25]. Our study showed significantly higher rates of prior CAZ-AVI and polymyxin exposure within 90 days among cefiderocol-resistant isolates, suggesting that these agents may indirectly diminish cefiderocol susceptibility by promoting porin alterations, β-lactamase variants, outer membrane modifications, or efflux pump activation.

Taken together, these findings underscore that even in the presence of high in vitro activity, the emergence of resistance in specific high-risk groups remains a concern requiring careful monitoring.

The main strengths of this study include its real-world design, the exclusive focus on BSIs—one of the most severe CRE-related syndromes—and the provision of baseline susceptibility data prior to the clinical availability of cefiderocol in Turkey. The study also delivers region-specific epidemiological insights from an OXA-48 endemic area with a rising prevalence of NDM co-producers. In resource-limited settings, where routine carbapenemase typing is often impractical due to cost and infrastructure limitations, identifying clinical predictors of resistance provides valuable guidance for empirical treatment decisions and stewardship efforts. Beyond microbiological outcomes, these findings contribute practical, real-world perspectives on anticipating resistance risk in clinical care and provide insights that are globally relevant, particularly in settings where carbapenemase typing is not routinely feasible.

Several limitations should be acknowledged. First, the single-center and retrospective design of this study restricts the generalizability of the findings. Second, carbapenemase genotyping was not systematically performed, limiting the ability to directly link resistance to molecular mechanisms. Third, the small number of resistant isolates precluded multivariable analysis. Most importantly, the study does not include patient outcome data, and its conclusions are confined to in vitro susceptibility results. This limitation is particularly relevant in light of previous reports suggesting higher rates of treatment failure and mortality with cefiderocol compared to the best available therapy in certain multidrug-resistant infections [26]. Therefore, while our findings provide important microbiological and epidemiological insights, they should not be interpreted as evidence of clinical efficacy.

## 4. Materials and Methods

### 4.1. Study Design and Setting

This retrospective, observational study included patients diagnosed with CRE-BSIs who were hospitalized between 1 January and 31 December 2023, at a tertiary university hospital in Istanbul, Turkey. The study protocol, case definitions, and inclusion/exclusion criteria were predefined and applied consistently to all patients, and all information was obtained from electronic medical records and the hospital information system.

### 4.2. Inclusion Criteria

Eligible patients met all of the following criteria:

- Adults aged ≥ 18 years;
- A diagnosis of a CRE-BSI confirmed by blood culture and antimicrobial susceptibility testing;
- Availability of antimicrobial susceptibility testing results for cefiderocol, CAZ-AVI, and colistin.

### 4.3. Exclusion Criteria

Patients were excluded if they had
Polymicrobial bloodstream infections;
Incomplete clinical or microbiological data.

### 4.4. Study Approval

- The study was approved by the Istanbul Medipol University Clinical Research Ethics Committee (date/number: 4 August 2025/E-10840098–202.3.02–4982).
- This retrospective observational study was conducted in accordance with the World Medical Association’s Declaration of Helsinki and the Ethical Principles for Medical Research Involving Human Subjects.
- The Non-invasive Clinical Research Ethics Committee of Medipol University waived the requirement for informed consent as the study was retrospective in design.

### 4.5. Data Collection

Demographic and clinical data included age, sex, major comorbidities (diabetes, heart failure, renal, rheumatologic, and neurological diseases), congenital or acquired immunodeficiency, hematologic/solid organ malignancy, and transplant history (SOT or HSCT). The dataset also captured CCI [27], prior CRE infection/colonization, recent antibiotic exposure (CAZ-AVI or polymyxins ≤ 90 days), hospitalization within the past year, neutropenia at culture, presence of invasive devices, ICU admission, sepsis or septic shock [28], and Pitt Bacteremia Score [29].

Microbiological data included the source of infection, classified as a primary or secondary BSI according to the Centers for Disease Control and Prevention/National Healthcare Safety Network (CDC/NHSN) definitions [30]. Secondary BSIs were further categorized as pneumonia, catheter-related BSIs, intra-abdominal infections, or urinary tract infections. Pathogen species and susceptibility results for cefiderocol, CAZ-AVI, and colistin were also recorded.

### 4.6. Microbiological Analysis

Blood cultures were processed using the BacT/ALERT 3D system (bioMérieux, Marcy-l’Étoile, France). Positive bottles were subcultured onto sheep blood agar, MacConkey agar, and chocolate agar (bioMérieux, Marcy-l’Étoile, France). Species identification was performed using Matrix-Assisted Laser Desorption/Ionization Time-of-Flight Mass Spectrometry (MALDI TOF MS) (*Bruker Daltonics, Bremen, Germany*).

Antimicrobial susceptibility testing was conducted according to the EUCAST 2023 guidelines [31]. Cefiderocol susceptibility was initially assessed by disk diffusion using Thermo Scientific™ Oxoid™ (Thermo Fisher Scientific, Waltham, MA, USA) Cefiderocol Antimicrobial Susceptibility Discs (30 µg). Results falling within the EUCAST-defined area of technical uncertainty (ATU) were then confirmed by broth microdilution using iron-depleted, cation-adjusted Mueller–Hinton broth (ID CAMHB, ComASP^®^ system). CAZ-AVI susceptibility was determined by Kirby–Bauer disk diffusion (*Oxoid, Basingstoke, UK*). Colistin MICs were determined by the reference broth microdilution method using the MIC COL kit (*Diagnostics, Galanta, Slovakia*).

### 4.7. Definitions

- CRE-BSI: Defined as the growth of an Enterobacterales isolate resistant to at least one carbapenem in blood culture [5].
- Sepsis and septic shock: Defined according to the Sepsis-3 criteria based on the Sequential Organ Failure Assessment (SOFA) score [28].
- Classification of BSIs: Determined according to CDC/NHSN definitions [30].
- Cefiderocol resistance: Determined according to the EUCAST 2023 [31] breakpoints. Isolates with inhibition zones < 17 mm were classified as resistant, while those with zones between 17 and 22 mm were considered within ATU and retested by broth microdilution; isolates with a minimum inhibitory concentration (MIC) > 2 µg/mL were defined as resistant [31].
- Colistin resistance: Defined as an MIC > 2 µg/mL according to the EUCAST criteria [31].
- CAZ-AVI resistance: Defined as an inhibition zone < 17 mm according to the EUCAST criteria [31].

### 4.8. Statistical Analysis

Statistical analyses were performed using IBM SPSS Statistics for Windows, version 26.0 (*IBM Corp., Armonk, NY, USA*). Continuous variables are expressed as the median (interquartile range, IQR), and categorical variables as frequencies and percentages. The Chi-squared test or Fisher’s exact test was used for categorical variables, and the Mann–Whitney U test for continuous variables. Paired comparisons of susceptibility rates for cefiderocol, CAZ-AVI, and colistin were performed using McNemar’s test. Risk factors for cefiderocol resistance were assessed through univariate analysis; multivariable logistic regression was not feasible due to the limited number of resistant isolates. A two-tailed *p*-value < 0.05 was considered statistically significant.

## 5. Conclusions

In this pre-implementation study, cefiderocol exhibited the highest in vitro activity among the tested agents against CRE-BSI isolates. Resistance, although infrequent, was observed. These exploratory observations may suggest potential links with clinical factors, such as hematologic malignancy, stem cell transplantation, prior antibiotic exposure, and prolonged hospitalization, but further studies are needed to confirm these associations. While our study did not include genotypic testing, these findings provide baseline microbiological data from a Turkish setting where OXA-48 predominates and NDM co-producers are increasingly reported in the literature. These findings provide baseline microbiological and epidemiological data and highlight the need to identify clinical predictors of resistance in settings lacking routine carbapenemase testing. Further multicenter and prospective studies are needed to expand on these observations and to inform clinical decision-making and future research in this field.

## Figures and Tables

**Table 1:** Demographic and clinical characteristics of patients with CRE bloodstream infections according to their cefiderocol susceptibility status.

Variable	Overall *n* = 202 (100%) ^1^	Cefiderocol-Resistant *n* = 12 (5.9%)	Cefiderocol-Susceptible *n* = 190 (94.1%)	*p*-Value ^2^
Age, median (IQR)	52.50 (35.75–70)	63.50 (42.25–74.5)	52.00 (35–70)	0.260
Age ≥ 65	68 (33.7)	6 (50)	62 (32.6)	0.217
Male sex	106 (52.5)	3 (25)	103 (54.2)	0.051
CCI, median (IQR)	5 (2–7)	2.5 (1.25–6)	5 (2–7)	0.134
CCI ≥ 4	124 (61.4)	5 (41.7)	119 (62.6)	0.148
Hematological malignancy	29 (14.4)	9 (75)	20 (10.5)	<0.001
HSCT	11 (5.4)	6 (50.0)	5 (2.6)	<0.001
Neutropenia	18 (8.9)	2 (16.7)	16 (8.4)	0.331
ICU	77 (38.1)	4 (33.3)	73 (38.4)	0.725
Invasive device	96 (47.5)	5 (41.7)	91 (47.9)	0.675
Sepsis	102 (50.5)	8 (66.7)	94 (49.5)	0.248
Septic shock	61 (30.2)	4 (33.3)	57 (30)	0.807
PBS, median (IQR)	4 (2–6)	4 (1–5.75)	4 (2–6)	0.890
Prior CRE infection	34 (16.8)	2 (16.7)	32 (16.8)	0.987
CRE colonization	33 (16.3)	1 (8.3)	32 (16.8)	0.439
Prior CAZ-AVI ≤ 90 days	50 (24.8)	10 (83.3)	40 (21.1)	<0.001
Prior polymyxin ≤ 90 days	53 (26.2)	9 (75)	44 (23.2)	<0.001
Hospitalization ≤ 1 year	71 (35.1)	9 (75)	62 (32.6)	0.003
LOS, median (IQR)	11 (7–16)	18 (15–22.25)	11 (7–15.25)	<0.001
LOS ≥ 14 days	80 (39.6)	11 (91.7)	69 (36.3)	<0.001

^1^ *n* (%) and median (IQR 25–75) results. ^2^ Pearson’s Chi-squared test; Mann–Whitney U test; Fisher’s exact test. IQR: interquartile range; CCI: Charlson Comorbidity Index; HSCT: hematopoietic stem cell transplantation; PBS: Pitt Bacteremia Score; ICU: intensive care unit; CRE: carbapenem-resistant Enterobacterales; CAZ-AVI: Ceftazidim avibactam; LOS: length of hospital stay.

**Table 2:** Microbiological data, including causative microorganism and type of BSI.

Variable	Overall *n* = 202 (100%) ^1^	Cefiderocol-Resistant *n* = 12 (5.9%)	Cefiderocol-Susceptible *n* = 190 (94.1%)	*p*-Value ^2^
**Source of Infection**				
Primary BSI	40 (19.8)	2 (16.7)	38 (20)	0.779
Secondary BSI	162 (80.2)	10 (83.3)	152 (80)	0.232
Pneumonia	50 (24.8)	4 (33.3)	46 (24.2)	
Catheter-related BSI	30 (14.9)	0 (0)	30 (15.8)	
Intra-abdominal infection	45 (22.3)	5 (41.7)	40 (21.1)	
Urinary tract infection	37 (18.3)	1 (8.3)	36 (18.9)	
**Type of CRE**				0.642
*Klebsiella pneumoniae*	190 (94.1)	11 (91.7)	179 (94.2)	
*Escherichia coli*	10 (5)	1 (8.3)	9 (4.7)	
*Enterobacter* spp.	2 (1)	0 (0)	2 (1.1)	

^1^ *n* (%). ^2^ Pearson’s Chi-squared test; Mann–Whitney U test; Fisher’s exact test. BSI: bloodstream infection; CRE: carbapenem-resistant Enterobacterales.

## Data Availability

The datasets generated during and/or analyzed during the current study are not publicly available due to administrative reasons, but are available from the corresponding author on reasonable request.
